# Soluble epoxide hydrolase inhibition alleviates chemotherapy induced neuropathic pain

**DOI:** 10.3389/fpain.2022.1100524

**Published:** 2023-01-09

**Authors:** Ashley A. Takeshita, Bruce D. Hammock, Karen M. Wagner

**Affiliations:** ^1^ EicOsis LLC, Davis, CA, United States; ^2^ Department of Entomology and Nematology and UC Davis Comprehensive Cancer Center, University of California Davis, Davis, CA, United States

**Keywords:** soluble epoxide hydrolase, chemotherapy induced peripheral neuropathy, neuropathic pain, analgesia, epoxy-fatty acids

## Abstract

Chemotherapy induced peripheral neuropathy (CIPN) is a particularly pernicious form of neuropathy and the associated pain is the primary dose-limiting factor of life-prolonging chemotherapy treatment. The prevalence of CIPN is high and can last long after treatment has been stopped. Currently, late in the COVID-19 pandemic, there are still increased psychological pressures on cancer patients as well as additional challenges in providing analgesia for them. These include the risks of nonsteroidal anti-inflammatory drug (NSAID) analgesics potentially masking early infection symptoms and the immunosuppression of steroidal and opiate based approaches. Even without these concerns, CIPN is often inadequately treated with few therapies that offer significant pain relief. The experiments we report use soluble epoxide hydrolase inhibitors (sEHI) which relieved this intractable pain in preclinical models. Doses of EC5026, an IND candidate intended to treat neuropathic pain, elicited dose dependent analgesic responses in multiple models including platinum-based, taxane, and vinca alkaloid-based CIPN pain in Sprague Dawley rats. At the same time as a class, the sEHI are known to result in fewer debilitating side effects of other analgesics, likely due to their novel mechanism of action. Overall, the observed dose-dependent analgesia in both male and female rats across multiple models of chemotherapy induced neuropathic pain holds promise as a useful tool when translated to the clinic.

## Introduction

Chemotherapy induced peripheral neuropathy (CIPN) is a painful condition that is particularly difficult to treat because it results from the on-target effects of chemotherapy, and the neuropathy often dose-limits essential anti-cancer treatment. The symptoms occur as bilateral, symmetrical distal sensations of burning or tingling pain with a sensitivity to touch and heat (1). The prevalence of CIPN has been reported to reach 68% in the first month after therapy (2) and the neuropathy from certain types of chemotherapeutic agents also lasts long after cessation of treatment (3–5). It can even develop and/or worsen after the cessation of treatment, an effect often referred to as coasting (6), and may require analgesic therapy after the end of chemotherapy regimens. Currently there are several mechanisms thought to contribute to the damage caused by chemotherapeutics of several classes that result in painful neuropathy. Antineoplastic drugs that directly affect microtubules such as taxanes and vinca alkaloids are thought to impact these processes in neurons as well as cancerous cells. However, painful neuropathy occurs in a similar fashion in many patients irrespective of the chemotherapeutic agent, which has led to broadening the hypothesized mechanisms of damage to include neuroinflammation (7), mitochondrial dysfunction (8, 9) and reactive oxygen species (ROS) production (10). The role of mitochondrial dysfunction in neuropathic pain has been established (11–13), and it has been demonstrated specifically for the painful CIPN neuropathy models used here (14, 15). Mitochondrial ATP production depends, in part, on the transmembrane potential of hydrogen ions which is formed by the mitochondrial membrane potential (ΔΨm), driven by proton pumps (Complexes I, III and IV), and the proton gradient (ΔpH). The ΔΨm plays a key role in mitochondrial homeostasis and is essential for the healthy functioning of cells (16). Chemotherapy agents including paclitaxel, vincristine, and oxaliplatin, among others, induce abnormal mitochondrial morphology in peripheral sensory nerves and dorsal root ganglia (DRG), reduce ΔΨm, and impair ATP production [reviewed in (17)]. Oxidative stress, with production of ROS and reactive nitrogen species (RNS) has also been demonstrated to contribute to CIPN in several models (18–20).

As mentioned, painful CIPN can be severe enough to dose limit lifesaving chemotherapy. However, most analgesics used to treat painful CIPN also have their own dose limiting side effects. For example, narcotics, which are sometimes used despite their limited efficacy, cause respiratory depression and also addiction. Other non-narcotic analgesics such as non-steroidal anti-inflammatory drugs (NSAIDs) are typically ineffective for neuropathy and gabapentinoids (gabapentin, pregabalin) have motor-impairing side effects and are ineffective in large subpopulations of patients. Duloxetine, a selective serotonin and norepinephrine reuptake inhibitor antidepressant (SSNRI) and first-line treatment for diabetic peripheral neuropathic pain, has demonstrated limited success for use against CIPN (21–23) but has the problem of serotonin withdrawal and other motor impairing side effects. Thus, there are no current therapies that offer adequate pain relief.

Here we use a strategy targeting the soluble epoxide hydrolase (sEH) enzyme as an analgesic mechanism to combat painful CIPN, which we explore from a therapeutic perspective. A direct comparison of sEH inhibitors (sEHI) to some of the above listed standard analgesic therapies has revealed superior analgesia with sEHI in neuropathic pain models (24). In addition, sEHI administration is not associated with the sedation and altered motor function produced by gabapentinoids. These side effects of gabapentinoids pose a risk especially for patients with secondary concerns of bone density and frailty who are prone to falling.

The sEHI technology is based on small molecule inhibitors of the sEH enzyme, a master regulator of epoxy-fatty acids (EpFAs) with a key role in the biology and pathogenesis of pain as well as many inflammation-driven chronic diseases. The sEHI EC5026 targets sEH which is downstream from the cytochrome P450 enzymes (CYP450s) in the arachidonic acid (ARA) cascade. Long chain polyunsaturated fatty acids such as ARA and docosahexaenoic acid (DHA) are transformed by CYP450 enzymes into EpFAs (for example, the epoxyeicosatrienoic acids, EETs, are formed from ARA). sEH degrades the anti-inflammatory and analgesic EpFAs to inactive or pro-inflammatory diols (i.e., EETs in to the dihydroxyeicosatrienoic acids, DHETs). The EpFAs s are potently analgesic, but short-lived molecules that are eliminated within seconds *in vivo*. Inhibition of sEH leads to the stabilization of EpFAs and increases their levels in both plasma and tissues. sEHI globally increases the levels of the EpFAs from several long chain polyunsaturated fatty acids such as adrenic acid and eicosapentaenoic acid (EPA) in addition to ARA and DHA, all of which are anti-hyperalgesic when administered to animals (25, 26). The EETs specifically have been demonstrated to prevent the translocation of NF-*κ*B into the nucleus which is the mechanistic basis of EpFAs anti-inflammatory activity (27, 28). The sEHI also oppose prostaglandin E_2_ (PGE_2_)-induced pain, a process downstream of cyclooxygenase -2 (COX-2) upregulation and therefore distinct from the action of NSAIDs and steroids (29). Importantly, EpFAs and inhibition of sEH have been demonstrated to limit mitochondrial dysfunction in several studies. EETs reduced the loss of ΔΨm and mitochondrial permeability transition pore (mPTP) opening (30), and increased mitochondrial superoxide dismutase leading to improved viability in human cells (31). A sEHI delayed the loss of ΔΨm due to anoxia-reoxygenation (32). In another study sEHI attenuated the fragmentation of mitochondria while increasing the expression of mitofusin-2 (Mfn2) and reducing dynamin-related protein 1 (Drp1), reversed the loss of ΔΨm and decreased both intracellular and mitochondrial ROS (33).

Inhibiting sEH also blocks several markers of endoplasmic reticulum stress (ER stress) (34, 35). Apoptotic responses occur when ER stress is excessive, prolonged, or insufficiently neutralized and is initiated through downstream pathways such as ER-associated protein degradation (ERAD) and C/EBP homologous protein (CHOP) (36). Phosphorylated PERK results in the phosphorylation of eukaryotic initiation factor 2 (eIF2α), activating transcription factor-4 (ATF4) activation, and transcription of CHOP. Inhibiting sEH maintains the EpFAs which block phosphorylation of these key sensor proteins and significantly decreases X-box binding protein 1 (XBP1s) and activating transcription factor-6 (ATF6) expression (which would otherwise lead to CHOP activation), thereby halting apoptosis (34, 37). In addition to this action against ER stress, sEHI also normalizes phospho-p38 and phospho-JNK, kinase mediators of neuropathic pain (34). A variety of biological signals can influence the ER stress pathway such as unfolded and misfolded proteins, high glucose, or ROS, which can be contributed by mitochondrial dysfunction and other sources. EpFAs additionally reduce the effects of ROS to stabilize mitochondria (38). Thus, they reduce cellular stress at multiple levels.

The safety, pharmacokinetics, and pharmacodynamic profile of a previous sEHI in humans is reported in Chen et al. 2012 (39). This report describes, AR9281, a sEHI that was tested through phase 1 clinical trial as a planned therapy for hypertension. This phase 1 study provided proof of safety of AR9281 in healthy human subjects. Separately, another sEHI, GSK2256294A, has been investigated in multiple phase I clinical trials by GlaxoSmithKline for a potential pulmonary indication with no signs of adverse events (40, 41).

The sEHI EC5026 is currently being developed by EicOsis to treat neuropathic pain in humans. Despite the availability of numerous types of analgesics, neuropathic pain remains insufficiently treated leading to patient suffering and reduced quality of life. In nonclinical rodent models of diabetic neuropathy, oral doses of sEHI have demonstrated potent analgesia against mechanical hypersensitivity (24). The sEHI was shown to outperform a higher dose of gabapentin, a first-line therapy for human diabetic neuropathic pain. This initial demonstration of efficacy against neuropathic pain was followed by preclinical results in the chronic constriction injury model (42). In this surgical model of painful neuropathy, the sEHI was again robustly analgesic (43). In contrast to sEHI, it is well known that highly selective COX-2 inhibitors (coxibs) are ineffective for chronic pain conditions (44) despite the long established analgesic effects of inhibiting cyclooxygenase enzymes in inflammatory conditions (45–47). Despite their inability to mediate analgesia in CIPN pain, the role of COX-2 upregulation in pro-carcinogenetic cellular responses has been recently described and COX-2 inhibitors have demonstrated antitumor activity (48, 49). Thus, it is likely that cancer patients may be concomitantly treated with COX-2 inhibitors or be using them after completing their chemotherapy regimens. Dual inhibition of sEH and cyclooxygenase enzymes has demonstrated anti-tumor activity by limiting tumor size and metastasis (50–53). These examples include a combination of sEHI and coxib, but also a designed dual ligand for the two enzymes, PTUPB. Recently, EC5026 in combination with EP4 antagonists, was shown to effectively limit the Révész effect of treatment generated debris stimulating tumor growth and metastasis (54). Given this background of sEHI successfully reducing neuropathic pain, the applicable cellular mechanisms of action, and the benefits of robust analgesia and antitumor action when combined with coxibs, we further characterized sEHI mediated analgesia by testing it in several models of painful CIPN.

## Materials and methods

### Animals

All included experiments were conducted in accordance with protocols approved by the Institutional Animal Care and Use Committee at Antibodies Inc. or the University of California Davis and adhered to the National Institutes of Health guide for the care and use of Laboratory animals. Great care was taken to reduce the number and minimize suffering of the animals used. Sprague–Dawley male and female rats (250 to 300 g; Charles River, Wilmington, MA, USA) were housed 2 per cage with free access to food and water. They were maintained under a 12 h light/dark cycle with controlled temperature and relative humidity. The study used 150 animals total which were randomly divided into groups using simple randomization of cages (2 rats per cage) generated with Microsoft Excel. Behavioral testing was also randomized among these groups during experiments with assays performed between 9:00 a.m. and 5:00 *p*.m. Scientists running the behavioral experiments were blinded to the treatment dosing protocol at the time of the tests.

### Chemicals

Chemotherapeutics including paclitaxel, oxaliplatin and vincristine (Fisher Scientific, Pittsburgh, PA) were administered intraperitoneally (i.p.) per the following dose regimens: paclitaxel 2 mg/kg i.p. four times on alternate days (1, 3, 5, 7), vincristine 0.1 mg/kg i.p. over 12 days, with two 5-day cycles and a 2-day break in between and, oxaliplatin once at 6 mg/kg i.p. The sEHI EC5026 and EC5029 treatments tested on the established CIPN models were formulated as true solutions in PEG400 (Sigma Aldrich, St. Louis MO) which was tested as the vehicle, and all compounds were administered by oral gavage. Morphine sulfate was formulated in saline (Fisher Scientific, Pittsburgh, PA) and administered by subcutaneous injection. Pregabalin (Tocris, Minneapolis, MN) was formulated in water and administered by oral gavage.

### Nociceptive assays

Rats were acclimated and tested for baseline responses before induction of CIPN models. All nociceptive measurements were acquired by observers who were blinded to the treatments. The rats were tested for the development of allodynia post chemotherapy regime with a von Frey assay using an electronic aesthesiometer with a rigid tip to assess mechanical withdrawal thresholds (PWTs). Rats with confirmed sensitivity where then randomized for treatments. The von Frey scores are reported as the grams needed to induce a hind paw withdrawal response per animal, averaged ± SEM per treatment group. Average pretreatment baseline von Frey gram scores ± SEM were 72.7 ± 1.7 oxaliplatin, 77.2 ± 1.6 paclitaxel, and 70.7 ± 1.2 vincristine including both male and female rats per model. The baseline scores represent pre-CIPN model naïve scores are not included in the figures as they vary in temporal length from the treatment days due to the model induction with different chemotherapy regimens. For the calculated results to assess treatments, the treatment score per time point was divided by the day of treatment CIPN baseline score and multiplied by 100 to obtain the percent of baseline measure for each rat. The scores were averaged ± SEM and reported per treatment group.

We assessed motor skills in naïve rats with the open field assay before induction of the CIPN models, after CIPN induction for a model baseline, and then on the day of treatment with compound on board. For the open field assay, rats were placed in an open-field arena (40W cm × 40L cm × 30H cm) of a 16-square grid, clear acrylic open top chamber and observed for 2 min continuously. Activity was assessed and scored as the sum of lines each animal crossed with both hind paws and number of rears as a function of time. Any abnormal gaiting was also noted if observed. The scores are a sum of the lines crossed with both hind paws and total number of rearing.

### Study design and data analysis

Individual animals were assessed as the experimental unit and groups containing both male and female subjects were compared per over a dose range with vehicle controls for each chemotherapy model. All data are expressed as the mean ± standard error of the mean (SEM) and analyzed using SigmaPlot software (San Jose, CA). Statistical significance was determined using Two-way Repeated Measures ANOVA; or where appropriate with Mann–Whitney *U*-test. *P* values less than 0.05 are reported as statistically significant. A priori sample sizes were determined before the study based on 0.05 alpha and power level set at 0.8. This allowed for smaller sample sets being statistically significant where there was a greater response difference between groups. Inclusion criteria for the experiments were >25% changes in nociceptive scores from baseline. The chemotherapy regimens in some cases adversely affected health of some rodents resulting in exclusion and euthanasia for humane endpoints, thus altering the uniformity in number of subjects per group.

## Results

### Single dose oral administration of EC5026

**Figure 1:**
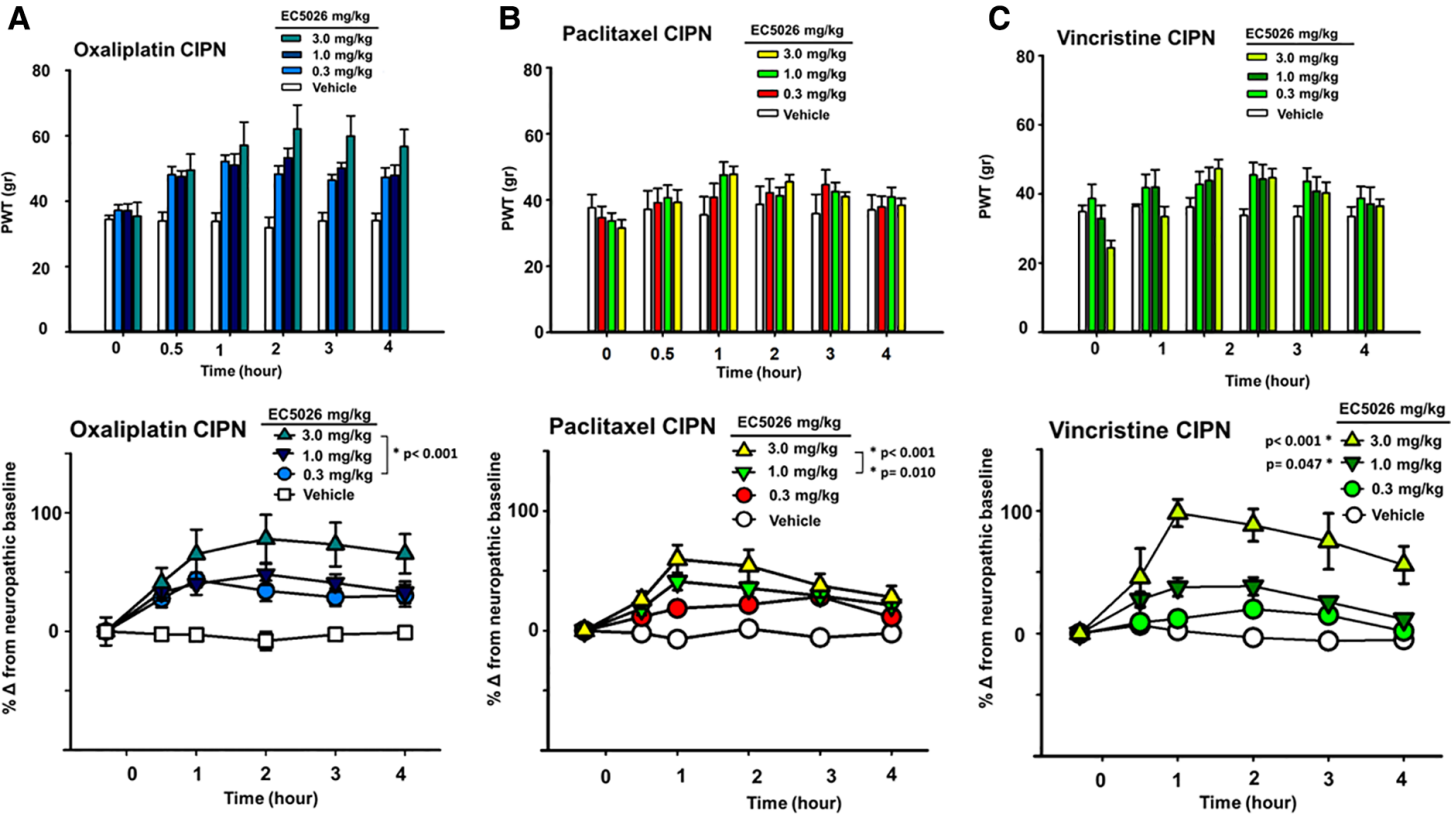
EC5026 efficacy in multiple CIPN models. In a group of Male and female Sprague Dawley rats EC5026 dose dependently improved mechanical paw withdrawal thresholds (PWT) in a von Frey assay. The gram scores for PWT were monitored for several hours (**A–C**, top figures) and the baseline painful scores which varied between treated groups were normalized to zero to assess and compare the percent improvement over time (**A–C**, bottom figures). The compared percent score results were analyzed using a Two-Way Repeated measures ANOVA for each model. (**A**) The doses from 0.3 to 3 mg/kg EC5026 administered by oral gavage significantly increased paw withdrawal thresholds in the oxaliplatin model (*p* < 0.001 all doses). (**B**) In the paclitaxel induced model the results of treatment were also significant compared to control in the 1 to 3 mg/kg dose range with EC5026 dose dependently improving PWT over time (*p* = 0.010 and *p* < 0.001 for 1 and 3 mg/kg respectively). (**C**) EC5026 was efficacious against vincristine induced painful CIPN, and the highest dose of EC5026 demonstrated a long duration of effect. (*p* = 0.047 and *p* < 0.001 for 1 and 3 mg/kg respectively).

The sEH inhibitor EC5026 was successful in relieving painful CIPN induced by several chemotherapeutics, all of which are currently used in clinical practice (Figure 1). Oral administration of EC5026 dose dependently increased the mechanical withdrawal thresholds of male and female CIPN rats compared to vehicle controls. The analgesia was observed in oxaliplatin, paclitaxel and vincristine induced CIPN pain in groups containing both male and female rats. The doses from 0.3 to 3 mg/kg administered by oral gavage significantly increased PWTs in the oxaliplatin model (Figure 1A, Two Way Repeated Measures ANOVA, Holm-Sidak *post hoc*, male and female rats *n* = 14 vehicle, *n* = 11 0.3 mg/kg, *n* = 16 mg/kg 1.0 mg/kg, *n* = 18 3.0 mg/kg, EC5026 treated groups vs. vehicle control, *p* < 0.001). In the paclitaxel induced model, the results were also significant compared to control with the 1 and 3 mg/kg doses of EC5026 dose dependently improving PWTs over time (Figure 1B, Two Way Repeated Measures ANOVA, Holm-Sidak *post hoc*, male and female rats *n* = 5 vehicle, *n* = 9 0.3 mg/kg, *n* = 12 mg/kg 1.0 mg/kg, *n* = 11 3.0 mg/kg, EC5026 treated groups vs. vehicle control, *p* = 0.010 and *p* < 0.001 for 1 and 3 mg/kg respectively). In the vincristine induced painful CIPN model EC5026 was efficacious and at the highest 3 mg/kg dose demonstrated a long duration of effect. (Figure 1C, Two Way Repeated Measures ANOVA, Holm-Sidak *post hoc*, male and female rats *n* = 5 vehicle, *n* = 7 0.3 mg/kg, *n* = 7 mg/kg 1.0 mg/kg, *n* = 8 3.0 mg/kg, EC5026 treated groups vs. vehicle control, *p* = 0.047 and *p* < 0.001 for 1 and 3 mg/kg respectively). Importantly for all models, there was a rapid effect with increased PWTs within 30 min to 1 h post oral administration and the effects lasted several hours in duration. There appeared to be difference in the magnitude of response between models with greater overall change in the oxaliplatin and vincristine models. The vincristine model was then employed further to investigate EC5026 in drug combinations and against standard-of-care positive controls.

### sEH inhibitor efficacy in combination with cyclooxygenase inhibitors

**Figure 2:**
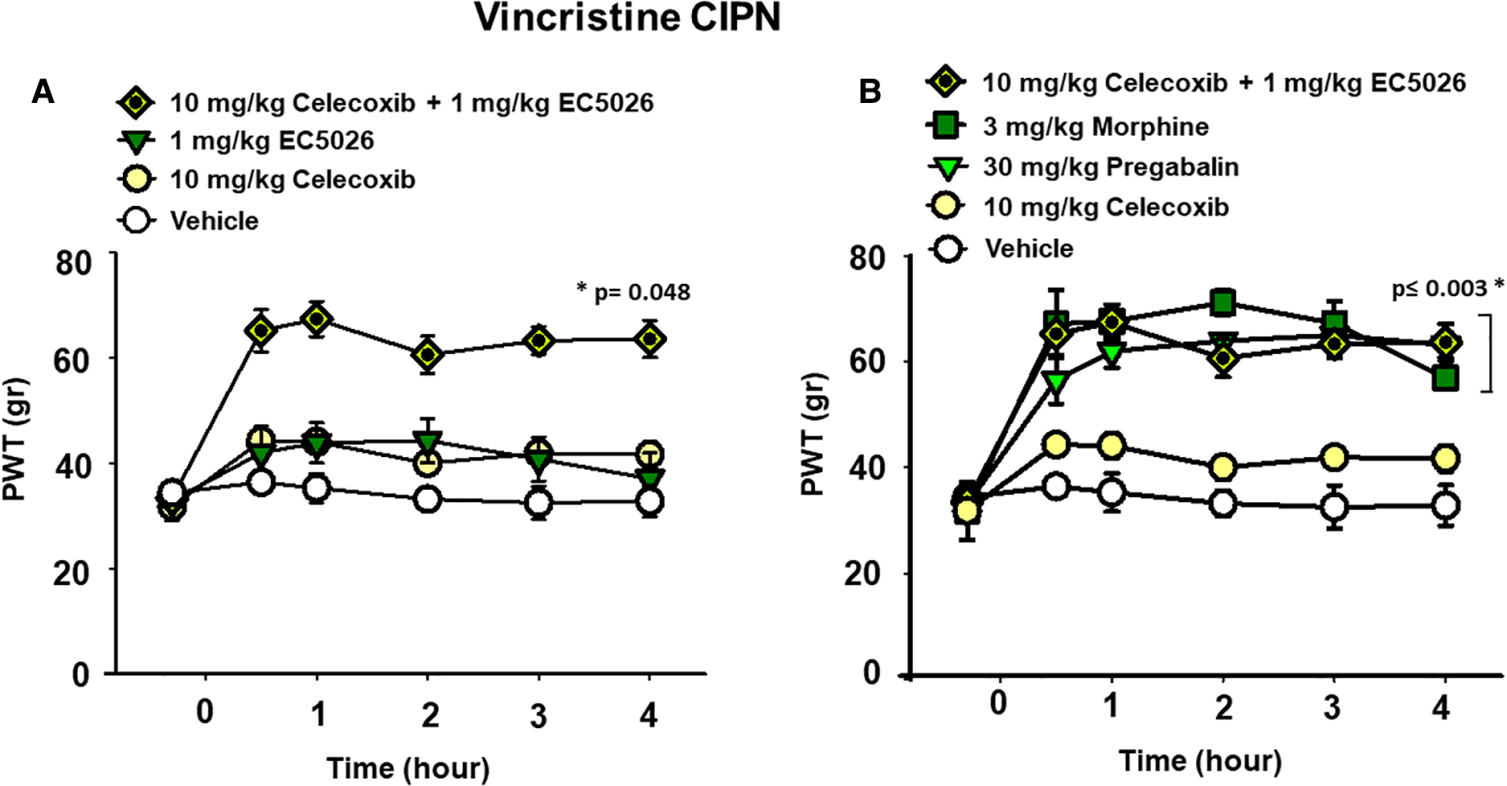
EC5026 in low dose combination with celecoxib is synergistically analgesic and outperforms standard of care therapies. (**A**) In the vincristine induced CIPN model a combination of 1 mg/kg EC5026, and 10 mg/kg celecoxib was robustly analgesic and had improved efficacy over either of the single administrations of EC5026 or celecoxib. The combination was able to increase paw withdrawal thresholds (*p* = 0.048) of grouped Male and female rats with neuropathic pain tested in the von Frey assay. (**B**) The combination of 1 mg/kg EC5026 and 10 mg/kg celecoxib was of similar efficacy to both 3 mg/kg morphine and 30 mg/kg of pregabalin in the same assay. These drugs significantly increased paw withdrawal thresholds (*p* ≤ 0.003) compared to vehicle control. As expected, the single administration of celecoxib was not effective against CIPN pain.

We investigated the potential effects of EC5026 in combination with celecoxib to evaluate analgesia, specifically, in the painful CIPN models. EC5026 (1 mg/kg) was combined with low dose celecoxib (10 mg/kg) orally dosed in the same solution. The combination far exceeded the efficacy of an individual dose of celecoxib in the CIPN model as well as vehicle controls. This was not unexpected given the lack of efficacy typically demonstrated by cyclooxygenase inhibitors in CIPN and the potency of sEHI in this model. However, the combination also exceeded the 1 mg/kg EC5026 single dose and demonstrated an apparent synergy in blocking mechanical allodynia in these animals. (Figure 2A Two Way Repeated Measures ANOVA, Holm-Sidak *post hoc*, male and female rats *n* = 5 vehicle, *n* = 7 EC5026 1 mg/kg, *n* = 6 celebrex 10 mg/kg, *n* = 4 EC5026 1 mg/kg + celebrex 10 mg/kg, treated groups vs. control, *p* = 0.048).

### Standard-of-care comparisons

A major standard-of-care therapy for painful chronic neuropathy is pregabalin. Although painful CIPN is not a listed indication for this drug, pregabalin and duloxetine are employed for pain relief in this condition. Opiates remain an option for breakthrough pain and are often administered despite their limitations for treating chronic pain. Doses of pregabalin (30 mg/kg) and morphine (3 mg/kg) were compared to the combination of EC5026 with celecoxib (1 + 10 mg/kg) (Figure 2B). The positive controls pregabalin and morphine and the EC5026 + celecoxib combination all significantly increased the PWTs compared to single administration of celecoxib and vehicle. The combination demonstrated potent efficacy that resulted in PWT scores that were not statistically different from either the 30 mg/kg pregabalin and 3 mg/kg morphine treatments. (Two Way Repeated Measures ANOVA, Holm-Sidak *post hoc*, male and female *n* = 5 vehicle, *n* = 4 EC5026 1 mg/kg + celebrex 10 mg/kg, *n* = 6 celebrex 10 mg/kg, *n* = 6 pregabalin 30 mg/kg, *n* = 4 morphine 3 mg/kg, pairwise analysis, *p* ≤ 0.003). Importantly, the combination had a rapid onset with long duration and was equipotent to the positive controls pregabalin and morphine that have well-known motor impairing side effects which are absent for sEHI. Open field assay scores indicated there was no change in motor skill or exploration with EC5026 at the highest dose tested in the CIPN models (Supplementary Figure S1).

### Analysis of sexual dimorphism in pain response

**Figure 3:**
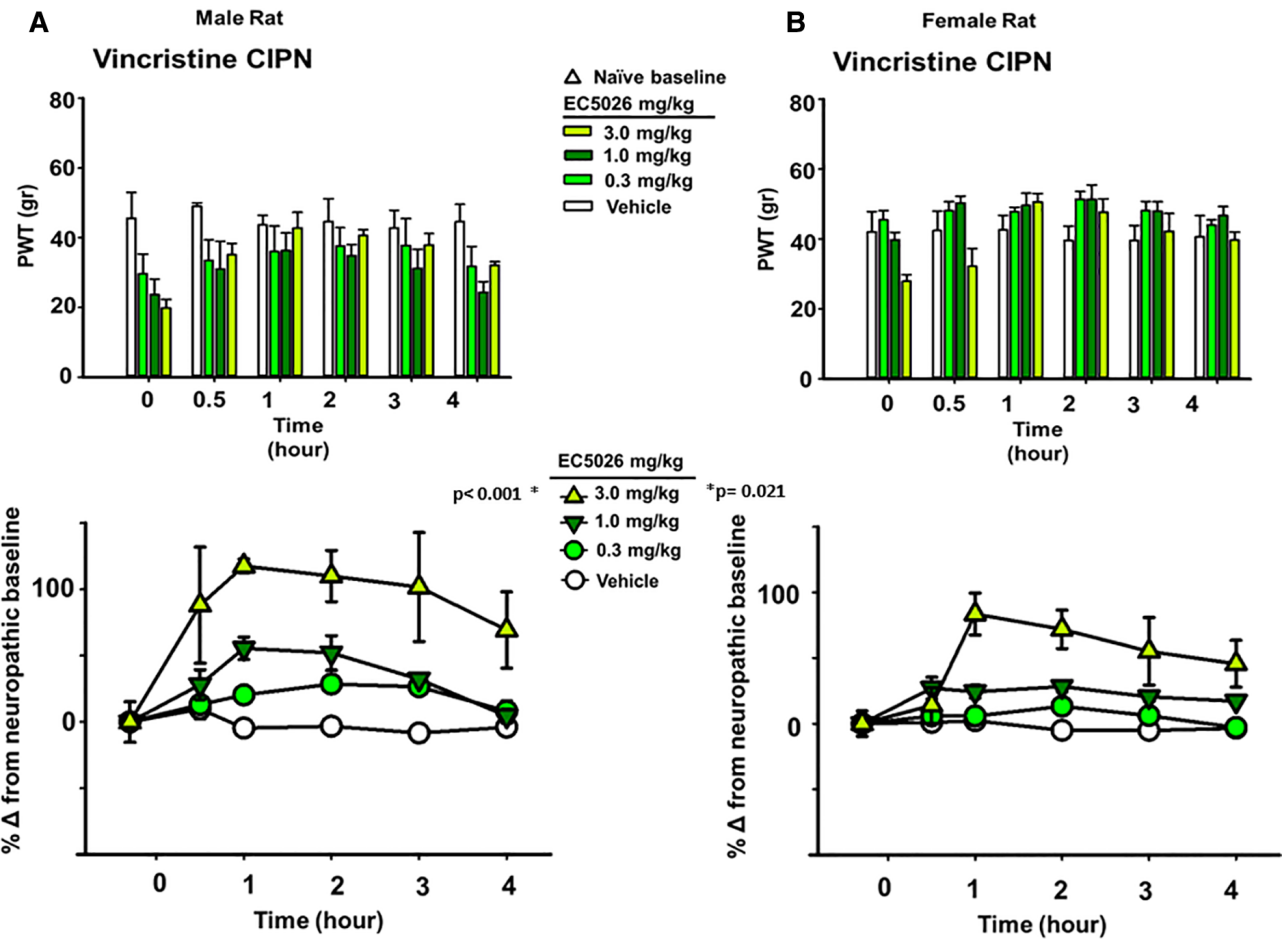
EC5026 is robustly analgesic in both Male and female rats with painful CIPN. The vincristine model was used to evaluate the analgesia in both Male and female animals. (**A**) The paw withdrawal threshold (PWT) raw scores in gram for males (top) show the increase over time with EC5026 oral administration. The normalization of the baselines to compare treatments (bottom) display the efficacy of the dose dependent analgesia. The gram scores do not match the original naïve scores; however, the improvement reaches to 100% over the CIPN painful state eliciting effective analgesia. (**B**) In female rats the PWT results (top) are similar to males but with somewhat higher CIPN baseline scores overall. Normalizing the CIPN baselines demonstrates the analgesic efficacy of the single administration of EC5026. Moreover, the dose dependent analgesia was effective in raising PWTs measured in the von Frey assay for both sexes.

The allodynia induced by the different chemotherapeutic agents did not alter the average painful condition between male and female rats. It has previously demonstrated, at least for the paclitaxel model in rats, that a sex-based difference is not observed in mechanical allodynia with induction of the neuropathic pain (55). We analyzed the vincristine model for sexual dimorphisms in PWT response because it had the largest observed magnitude of response to EC5026 among the three models (depicted in Figure 1). This analysis demonstrated that there was no statistical difference between the male or female rats in response to the EC5026 treatment over all included doses (Two Way Repeated Measures ANOVA, Holm-Sidak *post hoc*, *n* = 4 female, *n* = 4 male, pairwise analysis, N.S. *p* = 0.172 male vs. female rats, *p* < 0.001 male 3 mg/kg vs. vehicle and *p* = 0.021 female 3 mg/kg vs. vehicle). There was variation in the painful CIPN baselines across dose groups for both sexes (Figure 3 top, PWT gram data) as well as an observable and statistically significant increase in PWTs at the 3 mg/kg dose for both male and female rats (Figure 3 bottom, normalized percent scores). The only apparent difference was for females at the 1 mg/kg dose which had increased PWTs but lacked significance. However, when sexes were grouped together the increase in PWTs was significant (Figure 1C, bottom). In summary, EC5026 was able to increase PWTs effectively in both male and female rats and was analgesic across the CIPN models.

## Discussion

Painful CIPN remains a common adverse event that occurs due to the use of antineoplastic agents and can lead to extended infusion times, dose reduction, or in severe cases, treatment cessation. To date there are no existing treatments to prevent painful CIPN and the available analgesics have limited efficacy and/or utility. The effects of the chemotherapy agents have been well established in rodents with oxaliplatin inducing mechanical hyperalgesia, allodynia, and mitochondrial injury indicated by mitochondrial swelling and vacuolation in peripheral nerves without observable nerve degeneration which also occurs in paclitaxel treated rats (56–58). Mitochondrial damage and dysfunction seen with taxanes including paclitaxel (59, 60) is also a shared injury mechanism with vinca alkaloids such as vincristine (61). The DRG are uniquely susceptible to the effects of chemotherapeutics as they lie outside the blood brain barrier and express transporters that allow some of these drugs to accumulate (62). This mechanism of CIPN toxicity targets DRG due to uptake *via* organic-anion-transporting-polypeptides (OATP1B1 and OATP1B3). Blockade of the homologous murine OATP1B2 was able to inhibit paclitaxel induced CIPN (63) and OATP1B3 has been detected in several types of cancer cells (64). Blocking chemotherapy uptake was not the experimental design here as we tested the sEHI after the development of painful neuropathy for their analgesic activity. However, the potential of sEHI to limit drug transport *via* these transporters should be thoroughly investigated, especially if a preventative approach of their use is intended.

Once taken up into the cells, oxidative stress in neurons is also known to contribute to the pathophysiology of painful CIPN (65, 66). Recent preclinical studies have implicated nuclear factor erythroid-2-related factor 2 (Nrf2) and peroxisome proliferator-activated receptor gamma (PPAR*γ*) activation may have a role in limiting oxidative stress (67, 68), though there are cautions with targeting these nuclear receptors for clinical efficacy (69, 70). Other antioxidant approaches have also been tested in preclinical experiments including N-acetylcysteine and vitamin E among others (71) however, the current American Society of Clinical Oncology (ASCO) guidelines indicate these should not be offered to cancer patients for an evidence-based lack of benefit (72). Employing a different mechanism, inhibiting the sEH enzyme with small molecules elevates EpFAs which diminish cellular stress by reducing ROS and limiting ER stress. Previously, administration of sEHI in modeled diabetic neuropathic pain demonstrated the ability of the sEHI to elevate EpFA/diol ratios in spinal cord tissue as well as plasma levels (24). To explore the breadth of analgesia elicited by sEH inhibition and specifically EC5026, we tested the compound against painful CIPN in several models using a taxane (paclitaxel), vinca alkaloid (vincristine) and platinum-based therapy (oxaliplatin). The current study focused on the analgesia assessed with nociceptive assays and did not examine the oxylipin profile correlated with EC5026 administration. Despite this limitation, the results of these initial experiments demonstrate that oral administration of EC5026 effectively improves the mechanical allodynia induced by several chemotherapeutics. The efficacy of EC5026 as a single analgesic agent across multiple models is coupled with its demonstrated safety in Phase 1 clinical trials in healthy volunteers.

An additional experimental model that remains to be explored is the administration of sEHI with a protease inhibitor chemotherapeutic agent such as bortezomib. Because bortezomib is hypothesized to induce painful neuropathy through a mitochondrial stress mechanism (73, 74), the sEHI may also improve outcomes in combination with this therapy as well. The broad efficacy of sEHI as an analgesic class was well known, but it was nevertheless remarkable to discover potent analgesia of the candidate EC5026 in the CIPN models. Inhibiting sEH is known to reduce mitochondrial stress (35, 75, 76), and this may contribute to the effect seen in the current experiments.

Although drugs such as pregabalin have been found to have limited efficacy in some patients, at least one study demonstrated its complete lack of efficacy in patients with oxaliplatin induced CIPN (77). Even duloxetine, which has the most positive results to date against CIPN, does not provide adequate relief of symptoms ((78), reviewed in (79)). Moreover, there is no indication that these drugs have any mechanism for preventing the acquisition of the neurological damage (6). The mechanisms of limiting mitochondrial dysfunction and ER stress with sEH inhibition may actually alter the course of this damage, however this preventative use of sEHI remains to be thoroughly investigated. As mentioned, the experimental approaches employed here used the CIPN models with fully developed neuropathic pain to investigate therapeutic analgesia thus, prophylactic paradigms were not tested. We did confirm the mechanism of sEH inhibition mediating analgesia in the CIPN models by testing a second structurally related sEHI, EC5029 at 3 mg/kg (Supplementary Figure S1), which demonstrated potent analgesia in all three models. While these experiments tested therapeutic potential, there is still a need for a preventative treatment for CIPN given the recent negative clinical trial results from using calmangafodipir, a mitochondrial superoxide dismutase mimetic with calcium replacing the manganese of the contrast agent mangafodipir (80).

CIPN has symptoms that are generally categorized into two types of issues, the tingling burning pain sensations and also a loss of sensation, both of which occur *via* the same mechanisms. We specifically target the pain in CIPN because it is reported to be the most bothersome symptom (81), and the hypersensitivity is demonstrated and measured in preclinical species with mechanical PWTs. A common clinical complaint is cold allodynia particularly with oxaliplatin induced neuropathy. In this study we limited our investigation to innocuous stimuli-based assays in order to assess the analgesia over a repeated trial time course and compare the results to other previously published neuropathy models. Given the efficacy demonstrated against the tactile allodynia in current study, a further investigation into the effect of sEHI on cold allodynia is warranted. However, the amelioration of mitochondrial injury and dysfunction as well as ER stress should mitigate all the CIPN symptoms related to these mechanisms.

There appeared to be a high magnitude of response to EC5026 with greater overall change in the oxaliplatin and vincristine models. The efficacy of EC5026 in the vincristine model, specifically, may also relate to the known anti-inflammatory action of sEHI and EpFAs (82–84). Vincristine induced CIPN has correlated with increased expression of inflammatory markers and the upregulation of pro-inflammatory genes in microglia in addition to the established mechanisms of disrupted microtubule assembly and spindle formation (85, 86). The vincristine model was therefore employed further to investigate EC5026 in combination with celecoxib and against standard-of-care positive controls. Targeting inflammatory cytokines has also been pursued as an approach to ameliorate induced CIPN for other classes of chemotherapeutics including paclitaxel (87–89) and oxaliplatin (90). Because sEHI are anti-inflammatory in addition to being analgesic, we tested a limited antibody-dependent cell mediated cytotoxicity (ADCC) test to investigate if the sEHI would change effector cell action. The sEHI alone and in combination with a positive control did not alter effector cell activity in the assay (Supplementary Table S1). Antibody based chemotherapeutics are not commonly associated with CIPN in contrast to platinum-based drugs, however this demonstrates there is not an action on effector cells if the sEHI are ever considered for analgesic use in earlier treatment stages.

Other early generation sEHI inhibitors have previously demonstrated synergistic responses in combination with COX-2 inhibitors in preclinical models (91, 92). Moreover, the combination of EC5026 with a coxib does not affect the analgesia mediated by EC5026 and even appears to increase pain relief. There may be an opportunity to elicit more analgesia using combinations with higher doses than 1 mg/kg EC5026, but the results here suggest synergistic pain relief from the combination and higher doses may not be warranted. Importantly, the utility of sEHI in general, and EC5026 specifically, in combination with coxibs and a mechanistically related strategy using EP4 receptor antagonists may have benefits for cancer patients beyond the improved analgesia seen in these experiments.

Here, we tested the analgesic efficacy of EC5026 in groups of both male and female rats in all the included models of this study. The result of this experimental design was dose dependent and significant analgesia in all models. We further chose the vincristine model to evaluate this analgesia for any sexual dimorphism. Results from the vincristine induced CIPN model demonstrated that the analgesia reached nearly 100% over pretreatment painful baseline for both sexes. There were some differences in the response graphs with males seemingly having lower mechanical PWTs overall and more area under the response curve with EC5026 administration. We did not conduct a PK analysis of vincristine for these studies; however it is possible that a demonstrated sexually dimorphic difference in *P*-glycoprotein expression in rats (93) could affect a difference in the model induction and subsequent behavioral responses. The pharmacokinetics of EC5026 have been evaluated in rat (94), and at the included doses and formulation there is adequate exposure to elicit analgesic efficacy. The treatment responses per the raw PWT scores appear limited but they represent a large increase over painful CIPN baselines in gram score. Importantly, it has also been shown that in preclinical studies sEH inhibition does not result in narcotic-like euphoria or somnolence/dizziness as with gabapentinoids which may alter paw withdrawal ability (43, 95). To confirm the normal motor skill and exploration of animals treated with EC5026 in the CIPN models, rats were assessed using an open field assay and showed no changes (Supplementary Figure S2). This suggests that the von Frey PWTs are meaningful and are not due to a motor skill impairment in these withdrawal-based assays for nociceptive hypersensitivity. Relative to this point, EC5026 is also a compound that would pair with additional nonpharmacological interventions such as exercise and cutaneous neuro-stimulatory treatment (scrambler therapy) that have been found to be beneficial in limiting pain in limited trial (96, 97). In sum, the comparison of both sexes demonstrates most strongly that the analgesia mediated by EC5026 is significant and dose dependent for both groups.

The chemotherapeutics that are known to cause the highest percent of painful CIPN cases are still widely used to treat commonly occurring cancers and the personal cost of painful CIPN is still burdening patients who survive their cancer diagnosis and treatment. Inhibiting the sEH enzyme is a novel mechanism that holds promise to alleviate painful CIPN. sEHI are active against putative cellular pathologies of CIPN including mitochondrial dysfunction, ER stress and neuroinflammation and have demonstrated analgesic activity against several classes of chemotherapeutics used to model painful CIPN. The sEHI EC5026 has shown exceptional efficacy in these preclinical studies and good PK and tolerability in Phase I human clinical trials. sEHI are therefore poised to be an alternative option to treat this recalcitrant painful condition.

## Data Availability

The original contributions presented in the study are included in the article/**Supplementary Material**, further inquiries can be directed to the corresponding author.
