# Supplementary material for: Soluble epoxide hydrolase inhibition alleviates chemotherapy induced neuropathic pain
Source: Front Pain Res (Lausanne). 2023 Jan 9;3:1100524. doi: 10.3389/fpain.2022.1100524 (PMC9868926; doi:10.3389/fpain.2022.1100524)
Supplement: Supplementary file 1 [file Datasheet1.pdf]

Supplementary Material

Supplementary Figure 1.

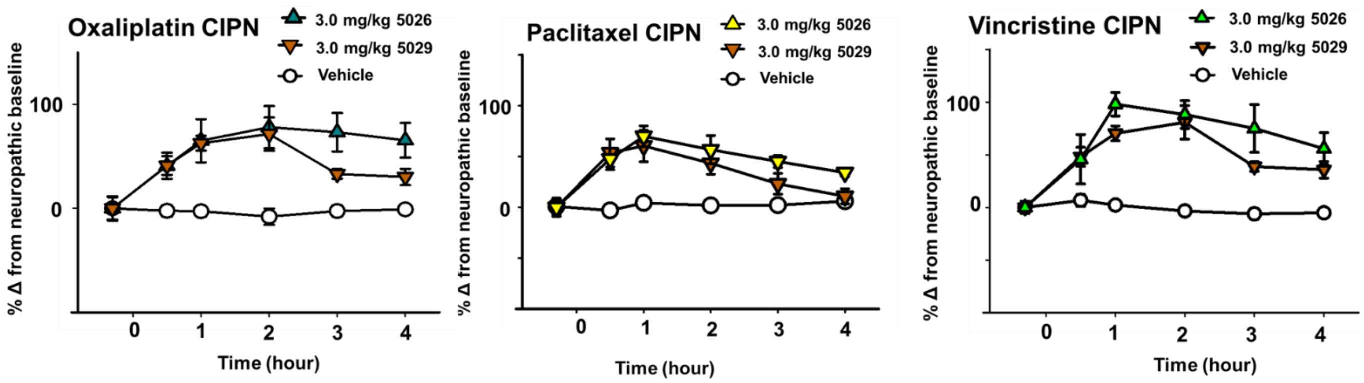

A second structurally related sEHI EC5029 was similarly efficacious against painful CIPN.

The most effective dose of EC5026 in these models including oxaliplatin, paclitaxel and vincristine was compared another sEH inhibitor EC5029, which revealed the potency of both compounds against this chronic pain. The sEHI were dosed in the same vehicle via oral gavage and followed the experimental paradigm in the nociceptive assays. While both compounds demonstrated good efficacy at a matched dose in groups of male and female rats, EC5026 appeared to be the more efficacious compound overall.

Supplemental Figure 2.

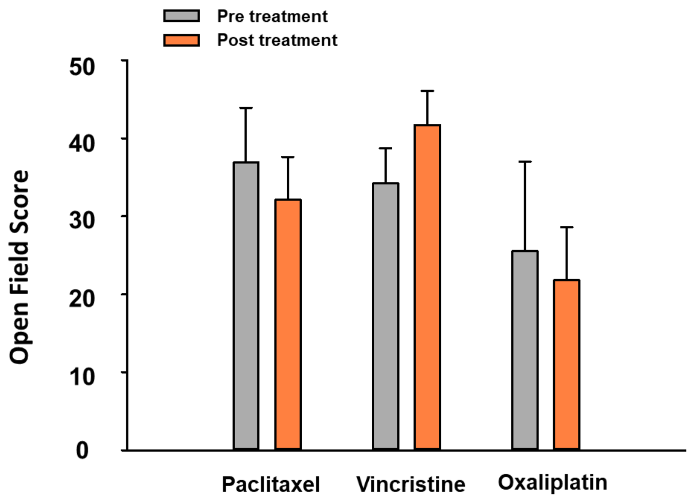

The sEHI do not impair exploration or motor function in the CIPN model.

EC5026 treatment at the highest 3 mg/kg dose did not alter the open field exploration or mobility of rats in any of the included CIPN models. All of the male and female rats with induced CIPN that were tested for nociceptive thresholds were also assessed for their pre-treatment open field scores and then at 1 hr post treatment with single oral dose of EC5026 (0.3 – 3.0 mg/kg dose range). There was no significant difference in the CIPN baselines (pre-treatment) compared to 1 the post-treatment scores in any of the models at the highest dose investigated.

Supplemental Table 1.

Target cell: SK-BR-3 **Effector cell:** Primary NK **E/T ratio:** 5:1

**Sample**	**EC50(µg/mL)**
Herceptin (positive control)	0.00008986
EC-5026	NA
EC-5026 (with 1ng/mL Herceptin)	NA
Human IgG1 (negative control)	NA

In a contracted antibody-dependent cell mediated cytotoxicity (ADCC) test conducted by GenScript ProBio (Piscataway, NJ) in a HER2+ breast cancer SK-BR-3 cell line, the combination of Herceptin with EC5026 did not alter the function of human natural killer (NK) cells purified from peripheral blood mononuclear cells (PBMC) as effector cells. The effector cell to target cell ratio (E/T) was 5:1 with an EC_{50} for Herceptin of 0.00008986 µg/mL and no activity observed for EC5026 up to the top

concentration of 10 µg/mL with a dilution factor of 10 and 7 doses done in triplicate or the EC5026 10 µg/mL/1ng/mL Herceptin combination.
